# Association between Hemoglobin Glycation Index and In-Hospital all-cause mortality of patients with Congestive Heart Failure: a retrospective study utilizing the MIMIC-IV database

**DOI:** 10.3389/fendo.2025.1475063

**Published:** 2025-03-19

**Authors:** Ling Sun, Jie Yuan, Tao Wang, Bin Ning, Qinghua Yuan

**Affiliations:** ^1^ Department of Cardiology, Fuyang Tumor Hospital, Fuyang, China; ^2^ Department of Cardiology, Fuyang People’s Hospital Affiliated to Anhui Medical University, Fuyang, China; ^3^ Consultancy Department, Hanyi Data Technology (Shenzhen) Co., Ltd, Shenzhen, China; ^4^ Department of Cardiology, The Seventh Affiliated Hospital of Sun Yat-sen University, Shenzhen, China

**Keywords:** congestive heart failure, hemoglobin glycation index, in-hospital mortality, prognosis, MIMIC-IV

## Abstract

**Background:**

The aim of this study was to explore the relationship between the hemoglobin glycation index (HGI) of Congestive Heart Failure (CHF) patients and their risk of mortality within 365 days.

**Patients and methods:**

The Medical Information Mart for Intensive Care (MIMIC-IV) database supplied the patient data for this study, which was categorized into quartiles based on the HGI. The primary endpoint was all-cause mortality within a 365-day period. Kaplan-Meier (K-M) analysis was utilized to compare this primary endpoint across the four aforementioned groups. The relationship between the HGI and the endpoint was examined using restricted cubic splines (RCS) and a Cox proportional hazards analysis.

**Results:**

A total of 985 patients were included in this study. HGI was significantly associated with 30 days mortality (15.9%; HR, 0.79; 95% CI, (0.67~0.92); P=0.003) and 60 days mortality (19.3%; HR, 0.83; 95% CI, (0.72~0.96); P=0.011) and 90 days mortality (22.1%; HR, 0.86; 95% CI, (0.75~0.99); P=0.031) and 365 days mortality (30.7%; HR, 0.97; 95% CI, (0.86~1.09); P=0.611) in patients with critical CHF in the completely adjusted Cox proportional risk model. RCS analysis revealed a U-shaped relationship between HGI and outcome events. KM curves survival analysis suggests a correlation between 30 days and 365 days mortality in HGI and CHF patients.

**Conclusions:**

A higher HGI has a more protective effect than a low HGI for patients with CHF and was directly associated with short-term mortality rates. These findings may be helpful in the management of patients with CHF.

## Introduction

Heart Failure (HF) is a prevalent and serious cardiovascular disease characterized by high incidence and mortality rates globally (1). Previous research indicates that approximately 40% of patients with HF also have diabetes, and HF is a significant contributor to morbidity and mortality in individuals with Type 2 Diabetes Mellitus (T2DM) (2). Early identification of individuals at high risk for HF is essential for timely intervention and the development of effective preventive strategies.

Blood glucose control has been shown to provide long-term cardiovascular benefits focusing on fasting blood glucose(FPG)and glycated hemoglobin (HbA1c) control (3). FPG alone has a low predictive value for cardiovascular outcomes. HbA1c is currently the most reliable measure for evaluating long-term blood glucose control (4, 5). HbA1c, utilized in the diagnosis and management of DM, reflects an individual’s average blood glucose over a three-month period and is currently the most commonly used surrogate marker of the effectiveness of glucose-lowering interventions (6, 7). HbA1c is not only utilized for the diagnosis and treatment of DM but also plays a significant role in the individualized management of patients with HF (8). However, the relationship between HbA1c and mortality in patients with both DM and HF has yielded conflicting results. Some cohort studies indicate that elevated HbA1c levels increase the risk of death in HF patients (9), while others suggest that higher HbA1c levels may reduce the risk of death (10, 11) Additionally, some studies have reported a U-shaped relationship between the two (12) Given that various factors, such as ethnicity, age, genetic variation, red blood cell (RBC) lifespan, iron deficiency, and anemia, can cause discrepancies between HbA1c and blood glucose levels, Hempe et al. first proposed the use of the Hemoglobin Glycation Index (HGI) to quantify individual variations in HbA1c levels (13). The HGI, which defined as is the difference between observed and predicted HbA1c, is derived from either mean or FPG and serves as a predictive tool for severe hypoglycemia and major adverse cardiovascular events (14).

Previous studies have indicated that the HGI is closely associated with adverse cardiovascular events (15, 16). However, both high and low levels of HGI have been linked to adverse outcomes in patients with CHF to varying degrees. Therefore, in the present study, the MIMIC-IV was database was utilized to construct linear regression equations to calculate the HGI and analyze the correlation between the HGI and adverse outcomes in patients with critical CHF.

## Methods

### Study participants

The Massachusetts Institute of Technology (MIT) and Beth Israel Deaconess Medical Center (BIDMC) collaborated in developing the Medical Information Mart for Intensive Care (MIMIC-IV) electronic database (version 2.2), which was used in the present study. Qinghua Yuan, one of the study’s authors, completed the National Institutes of Health’s web-based course ‘Protecting Human Research Participants’ (Record ID: 13111980). Following this, access to the database for data extraction was granted to him. To safeguard patient privacy, the data were de-identified. Consequently, the ethical committee of Beth Israel Deaconess Medical Center waived the requirement for obtaining patients’ informed consent.

**Figure 1:**
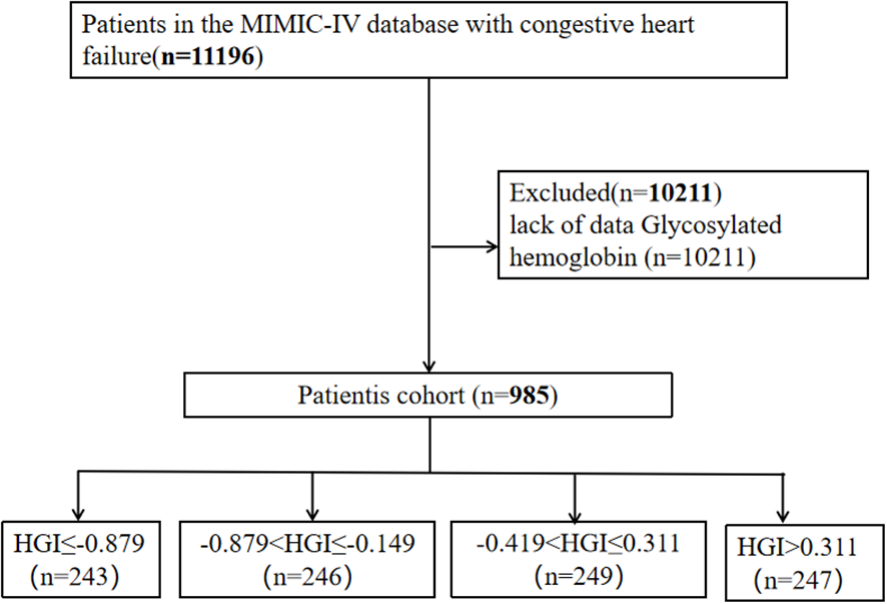
Flowchart of patient selection.

These 11196 patients were admitted to the intensive care unit (ICU) for the first time between 2008 and 2019 with CHF. We excluded 10211 patients lacking HbA1c level data; ultimately, 985 patients with severe CHF were eligible for the current study (**Figure 1**).

The exclusion criteria were as follows: (1) <18years of age, (2) acute coronary syndrome, (3) congenital heart disease, (4) advanced cancer, (5) lack of data at admission and (6) loss to follow-up.

### Data extractions

The software Postgres Structured Query Language (SQL) (version 13.7.2) and Navicate Premium (version 16) were used to extract information with a running SQL. The potential confounding variables included in this study are as follows: 1. Baseline demographic information: age, gender, race; vital signs: heart rate, systolic blood pressure (SBP), respiratory rate; 2. Comorbidities: Charlson Comorbidity Index (CCI), myocardial infarction (MI), cerebrovascular disease, diabetes, paraplegia, renal disease; 3. Laboratory parameters: white blood cells (WBC), RBC, hemoglobin (HGB), first creatinine.

### The definition of variable exposure and outcome events

Diabetes was diagnosed based on criteria established by the American Diabetes Association (17), which included a fasting plasma glucose level equal to or exceeding 126 mg/dL, a 2 h plasma glucose during OGTT equal to or exceeding 200 mg/dL, or an HbA1c level in whole blood equal to or exceeding 6.5%.

In this study, a linear regression model was established between FPG and HbA1c for all patients. Based on this model, the predicted HbA1c was calculated using the formula: Predicted HbA1c = 0.01×FPG (mg/dL) + 5.04. The difference between the measured and predicted HbA1c values is denoted as HGI (18). Four subgroups were established based on HGI quartiles: quartile 1 (Q1), (n=243, HGI ≤ -0.879; quartile 2 (Q2), n=246, -0.879<HGI≤-0.149; and quartile 3 (Q3), n=249, -0.419<HGI ≤ 0.311; and quartile 4 (Q4), n=247, HGI >0.311.

### Outcomes

The main objective of this study was 1-year mortality. Secondary outcomes focused on within 30 days, 60 days and 90 days all-cause mortality.

### Statistical analysis

All analyses were performed with the R statistical software package (http://www.R-project.org, R Foundation) and the Fengrui statistical software version 1.9.2 were used for all analyses. Categorical data were represented as percentages and statistically compared between groups via the chi-square test. Continuous numerical variables, following an assessment for normal distribution, were depicted as medians with their respective interquartile ranges and were compared across the quartiles employing the non-parametric Kruskal-Wallis test. The Cox proportional hazards model was employed to assess the HR associated with the HGI as a predictor of the outcome event, using the first quartile as the reference group. In the multivariate Cox regression analysis, factors such as age, gender, SBP, cerebrovascular disease, respiratory rate, CCI, WBC and RBC were considered as potential confounders. These variables were included in the model and underwent trend analysis to account for their influence on the results.

Kaplan-Meier (K-M) survival analysis was performed, stratifying the study population by quartile of the HGI, with the log-rank test used to evaluate differences among these groups. We investigated the relationship between HGI and the outcome event using restricted cubic splines (RCS) to identify threshold effects and inflection points within the HGI distribution. Additionally, subgroup analyses were conducted to validate our findings. The software PostgresSQL (version 13.7.2) and Navicate Premium (version 16) were used to extract information with a running SQL. A P-value of less than 0.05, in a two-tailed test, was considered to indicate statistical significance.

## Results

### Baseline information

The study included a total of 985 patients with CHF, comprising 579 male patients (58.8%) and 406 female patients (41.2%). Average age of all patients is 70.8 ± 14.0 years. Among them, 406 patients experienced fatal events during the 365-day follow-up period. The baseline data based on the HGI quartiles are presented in **Table 1**. Age, race, MI, cerebrovascular disease, diabetes, paraplegia, renal disease, CCI, SBP mean, resp rate mean, WBC, RBC and creatinine were significantly different among CHF. Gender and hemoglobin were not significantly different in **Table 1**.

**Table 1:** Basic characteristics of congestive heart failure with quartile of HGI.

Variables	Total (n = 985)	HGI≤-0.879 (n = 243)	-0.879<HGI≤-0.149 (n = 246)	-0.419<HGI ≤ 0.311 (n = 249)	HGI>0.311 (n = 247)	p	statistic
Gender, n (%)						0.142	5.441
1	579 (58.8)	141 (58)	156 (63.4)	133 (53.4)	149 (60.3)		
2	406 (41.2)	102 (42)	90 (36.6)	116 (46.6)	98 (39.7)		
age, Mean ± SD	70.8 ± 14.0	70.4 ± 14.0	72.4 ± 15.6	71.9 ± 13.5	68.4 ± 12.6	0.006	4.194
Race, n (%)						0.002	26.037
1	609 (61.8)	143 (58.8)	157 (63.8)	168 (67.5)	141 (57.1)		
2	97 (9.8)	19 (7.8)	15 (6.1)	25 (10)	38 (15.4)		
3	52 (5.3)	13 (5.3)	9 (3.7)	16 (6.4)	14 (5.7)		
4	227 (23.0)	68 (28)	65 (26.4)	40 (16.1)	54 (21.9)		
myocardial infarct, n (%)						0.019	9.988
0	517 (52.5)	107 (44)	133 (54.1)	135 (54.2)	142 (57.5)		
1	468 (47.5)	136 (56)	113 (45.9)	114 (45.8)	105 (42.5)		
cerebrovascular disease, n (%)						0.021	9.685
0	665 (67.5)	181 (74.5)	151 (61.4)	166 (66.7)	167 (67.6)		
1	320 (32.5)	62 (25.5)	95 (38.6)	83 (33.3)	80 (32.4)		
Diabetes, n (%)						< 0.001	300.647
0	542 (55.0)	173 (71.2)	198 (80.5)	148 (59.4)	23 (9.3)		
1	443 (45.0)	70 (28.8)	48 (19.5)	101 (40.6)	224 (90.7)		
paraplegia, n (%)						0.006	12.593
0	822 (83.5)	205 (84.4)	188 (76.4)	213 (85.5)	216 (87.4)		
1	163 (16.5)	38 (15.6)	58 (23.6)	36 (14.5)	31 (12.6)		
renal disease, n (%)						0.012	10.958
0	658 (66.8)	154 (63.4)	180 (73.2)	174 (69.9)	150 (60.7)		
1	327 (33.2)	89 (36.6)	66 (26.8)	75 (30.1)	97 (39.3)		
charlson comorbidity index, Mean ± SD	7.4 ± 2.6	7.2 ± 2.5	7.2 ± 2.8	7.5 ± 2.3	7.9 ± 2.4	0.005	4.239
sbp mean, Mean ± SD	121.2 ± 19.1	117.5 ± 21.0	122.6 ± 18.2	121.9 ± 18.1	122.7 ± 18.4	0.007	4.083
resp rate mean, Mean ± SD	20.3 ± 3.6	20.8 ± 3.6	20.4 ± 3.7	19.8 ± 3.6	20.4 ± 3.7	0.02	3.277
first white blood cells, Mean ± SD	12.0 ± 6.1	13.7 ± 6.7	11.8 ± 5.5	10.8 ± 4.6	11.6 ± 7.1	< 0.001	9.597
first hemoglobin, Mean ± SD	11.7 ± 2.3	11.4 ± 2.5	11.8 ± 2.2	11.6 ± 2.2	11.8 ± 2.1	0.223	1.465
first red blood cells, Mean ± SD	3.9 ± 0.8	3.8 ± 0.8	4.0 ± 0.7	4.0 ± 0.7	4.1 ± 0.7	< 0.001	6.604
first creatinine, Mean ± SD	1.6 ± 1.6	1.9 ± 1.9	1.4 ± 1.4	1.6 ± 1.6	1.6 ± 1.3	0.011	3.747

Gender 1: male, 2: female; Race 1: Yellow people, 2: White people, 3: black people, 4: other races.

### Mortality rate

Mortality rate of patients with CHF at different time periods after admission. The rate of mortality within 30 days was 15.9%, or 60 days was 19.3%, 90 days was 22.1%, and 365 days was 30.7% in **Table 2**.

**Table 2:** Mortality rate of patients with congestive heart failure at different time periods after admission.

Variables	Total (n = 985)	1 (n = 243)	2 (n = 246)	3 (n = 249)	4 (n = 247)	p	statistic
mor icu, n (%)						0.04	8.294
0	904 (91.8)	213 (87.7)	226 (91.9)	235 (94.4)	230 (93.1)		
1	81 (8.2)	30 (12.3)	20 (8.1)	14 (5.6)	17 (6.9)		
mor hospital, n (%)						0.071	7.041
0	864 (87.7)	202 (83.1)	220 (89.4)	225 (90.4)	217 (87.9)		
1	121 (12.3)	41 (16.9)	26 (10.6)	24 (9.6)	30 (12.1)		
mor 28d, n (%)						0.024	9.467
0	832 (84.5)	195 (80.2)	201 (81.7)	221 (88.8)	215 (87)		
1	153 (15.5)	48 (19.8)	45 (18.3)	28 (11.2)	32 (13)		
mor 30d, n (%)						0.012	10.96
0	828 (84.1)	192 (79)	201 (81.7)	221 (88.8)	214 (86.6)		
1	157 (15.9)	51 (21)	45 (18.3)	28 (11.2)	33 (13.4)		
mor 60d, n (%)						0.027	9.178
0	795 (80.7)	183 (75.3)	195 (79.3)	213 (85.5)	204 (82.6)		
1	190 (19.3)	60 (24.7)	51 (20.7)	36 (14.5)	43 (17.4)		
mor 90d, n (%)						0.052	7.714
0	767 (77.9)	176 (72.4)	189 (76.8)	205 (82.3)	197 (79.8)		
1	218 (22.1)	67 (27.6)	57 (23.2)	44 (17.7)	50 (20.2)		
mor 365d, n (%)						0.537	2.176
0	683 (69.3)	161 (66.3)	172 (69.9)	180 (72.3)	170 (68.8)		
1	302 (30.7)	82 (33.7)	74 (30.1)	69 (27.7)	77 (31.2)		

HGI, Hemoglobin Glycation Index.

Among the patients who died from CHF, HGI≤-0.879: 51 (21%) died within 30 days, 60 (24.7%) died within 60 days, 67 (27.6%) died within 90 days, and 82 (33.7%) died within 365 days. -0.879<HGI≤-0.149: 45 (18.3%) died within 30 days, 51 (20.7%) died within 60 days, 57 (23.2%) died within 90 days, and 74 (30.1%) died within 365 days. -0.419<HGI ≤ 0.311: 28 (11.2%) died within 30 days, 36 (14.5%) died within 60 days, 44 (17.7%) died within 90 days, and 69 (27.7%) died within 365 days. HGI>0.311: 33 (13.4%) died within 30 days, 43 (17.4%) died within 60 days, 50 (20.2%) died within 90 days, and 77 (31.2%) died within 365 days (**Tables 2**–**6**).

**Table 3:** Mortality rate within 30 days of admission in CHF patients with different HGI levels.

Variable	n.total	n.event_%	Followup.Time	crude.HR (95%CI)	crude.P value	adj.HR (95%CI)	adj.P value
HGI≤-0.879	243	51 (21)	6189.8	1 (Ref)		1 (Ref)	
-0.879<HGI≤-0.149	246	45 (18.3)	6474.3	0.84 (0.56~1.26)	0.405	0.86 (0.57~1.32)	0.493
-0.419<HGI ≤ 0.311	249	28 (11.2)	6920.7	0.5 (0.31~0.79)	0.003	0.54 (0.34~0.87)	0.012
HGI>0.311	247	33 (13.4)	6742.2	0.6 (0.39~0.93)	0.023	0.54 (0.32~0.89)	0.016
Trend.test	985	157 (15.9)	26327	0.81 (0.7~0.93)	0.004	0.79 (0.67~0.92)	0.003

HGI, Hemoglobin Glycation Index.

**Table 4:** Mortality rate within 60 days of admission in CHF patients with different HGI levels.

Variable	n.total	n.event_%	Followup.Time	crude.HR (95%CI)	crude.P value	adj.HR (95%CI)	adj.P value
HGI≤-0.879	243	60 (24.7)	11821.8	1 (Ref)		1 (Ref)	
-0.879<HGI≤-0.149	246	51 (20.7)	12386	0.81 (0.56~1.18)	0.276	0.83 (0.56~1.22)	0.34
-0.419<HGI ≤ 0.311	249	36 (14.5)	13425.1	0.54 (0.36~0.82)	0.003	0.59 (0.38~0.9)	0.014
HGI>0.311	247	43 (17.4)	12977.1	0.66 (0.45~0.98)	0.04	0.63 (0.4~0.99)	0.043
Trend.test	985	190 (19.3)	50610	0.84 (0.74~0.96)	0.009	0.83 (0.72~0.96)	0.011

HGI, Hemoglobin Glycation Index; ICU, Intensive Care Unit.

**Table 5:** Mortality rate within 365 days of admission in CHF patients with different HGI levels.

Variable	n.total	n.event_%	Followup.Time	crude.HR (95%CI)	crude.P value	adj.HR (95%CI)	adj.P value
HGI≤-0.879	243	67 (27.6)	17178.8	1 (Ref)		1 (Ref)	
-0.879<HGI≤-0.149	246	57 (23.2)	18136.6	0.81 (0.57~1.15)	0.244	0.83 (0.57~1.21)	0.332
-0.419<HGI ≤ 0.311	249	44 (17.7)	19683.6	0.59 (0.4~0.86)	0.006	0.65 (0.44~0.96)	0.032
HGI>0.311	247	50 (20.2)	18948.2	0.69 (0.48~0.99)	0.045	0.68 (0.45~1.05)	0.083
Trend.test	985	218 (22.1)	73947.2	0.86 (0.76~0.97)	0.014	0.86 (0.75~0.99)	0.031

HGI, Hemoglobin Glycation Index.

**Table 6:** Mortality rate within 365 days of admission in CHF patients with different HGI levels.

Variable	n.total	n.event_%	Followup.Time	crude.HR (95%CI)	crude.P value	adj.HR (95%CI)	adj.P value
HGI≤-0.879	243	82 (33.7)	63642.8	1 (Ref)		1 (Ref)	
-0.879<HGI≤-0.149	246	74 (30.1)	67734.3	0.86 (0.62~1.17)	0.329	0.86 (0.62~1.19)	0.36
-0.419<HGI ≤ 0.311	249	69 (27.7)	71915.2	0.75 (0.54~1.03)	0.079	0.8 (0.58~1.12)	0.196
HGI>0.311	247	77 (31.2)	68698.3	0.87 (0.64~1.18)	0.368	0.97 (0.67~1.39)	0.849
Trend.test	985	302 (30.7)	271990.6	0.94 (0.85~1.05)	0.271	0.97 (0.86~1.09)	0.611

HGI, Hemoglobin Glycation Index.

### Correlation of the HGI with outcome events

In the comparison of patients’ baseline information, we found that the Q1 group (HGI≤-0.879) had the highest mortality rate compared to the remaining groups. Based on the above, we analyzed the correlation between HGI and the primary outcome by developing Cox proportional risk models with the Q1 group as the reference group. HGI was significantly associated with 30 days mortality (HGI 3: HR, 0.54; P= 0.012; HGI 4: HR, 0.54; P=0.016) and 60 days mortality (HGI 3: HR, 0.59; P=0.014 and HGI 4: HR, 0.63; P=0.043) and 90 days mortality (HGI 3: HR, 0.65; P=0.032) in patients with critical CHF in the completely adjusted Cox proportional risk model (**Table 7**).

**Table 7:** 30, 60, 90, 365days Cox regression of congestive heart failure with quartile of HGI.

	30 days		60 days		90 days		365 days	
Variable	adj.HR (95%CI)	adj.P value	adj.HR (95%CI)	adj.P value	adj.HR (95%CI)	adj.P value	adj.HR (95%CI)	adj.P value
-0.879<HGI≤-0.149	0.86 (0.57~1.32)	0.493	0.83 (0.56~1.22)	0.34	0.83 (0.57~1.21)	0.332	0.86 (0.62~1.19)	0.36
-0.419<HGI ≤ 0.311	0.54 (0.34~0.87)	0.012	0.59 (0.38~0.9)	0.014	0.65 (0.44~0.96)	0.032	0.8 (0.58~1.12)	0.196
HGI>0.311	0.54 (0.32~0.89)	0.016	0.63 (0.4~0.99)	0.043	0.68 (0.45~1.05)	0.083	0.97 (0.67~1.39)	0.849
Gender	1.24 (0.88~1.74)	0.22	1.16 (0.85~1.58)	0.342	1.3 (0.97~1.74)	0.078	1.16 (0.91~1.49)	0.224
age	1.03 (1.01~1.05)	0.001	1.03 (1.01~1.04)	<0.001	1.03 (1.01~1.04)	<0.001	1.02 (1.01~1.04)	<0.001
sbp mean	0.98 (0.97~0.99)	<0.001	0.98 (0.97~0.99)	<0.001	0.98 (0.97~0.99)	<0.001	0.99 (0.98~0.99)	<0.001
first white blood cells	1.03 (1.02~1.04)	<0.001	1.03 (1.01~1.04)	<0.001	1.03 (1.02~1.04)	<0.001	1.02 (1.01~1.04)	<0.001
first red blood cells	1.02 (0.81~1.29)	0.862	1.06 (0.86~1.31)	0.591	1.06 (0.87~1.29)	0.59	0.99 (0.84~1.18)	0.953
cerebrovascular disease	2.26 (1.53~3.35)	<0.001	2.32 (1.63~3.3)	<0.001	2.23 (1.6~3.1)	<0.001	1.75 (1.32~2.31)	<0.001
Diabetes	1.23 (0.83~1.83)	0.299	1.16 (0.81~1.66)	0.415	1.05 (0.75~1.47)	0.789	0.95 (0.71~1.27)	0.719
charlson comorbidity index	1.1 (1.01~1.19)	0.02	1.12 (1.04~1.2)	0.002	1.13 (1.06~1.21)	<0.001	1.15 (1.09~1.22)	<0.001
Race2	0.9 (0.45~1.82)	0.774	0.9 (0.48~1.69)	0.735	1.33 (0.8~2.18)	0.269	1.19 (0.79~1.81)	0.409
Race3	1.1 (0.48~2.53)	0.827	1.23 (0.59~2.54)	0.579	1.07 (0.52~2.21)	0.846	0.75 (0.38~1.47)	0.397
Race4	1.6 (1.11~2.29)	0.011	1.78 (1.29~2.46)	0.001	1.67 (1.23~2.29)	0.001		

Gender 1: male, 2: female; Race 1: Yellow people, 2: White people, 3: black people, 4: other races.

### Kaplan-Meier survival analysis

**Figure 2:**
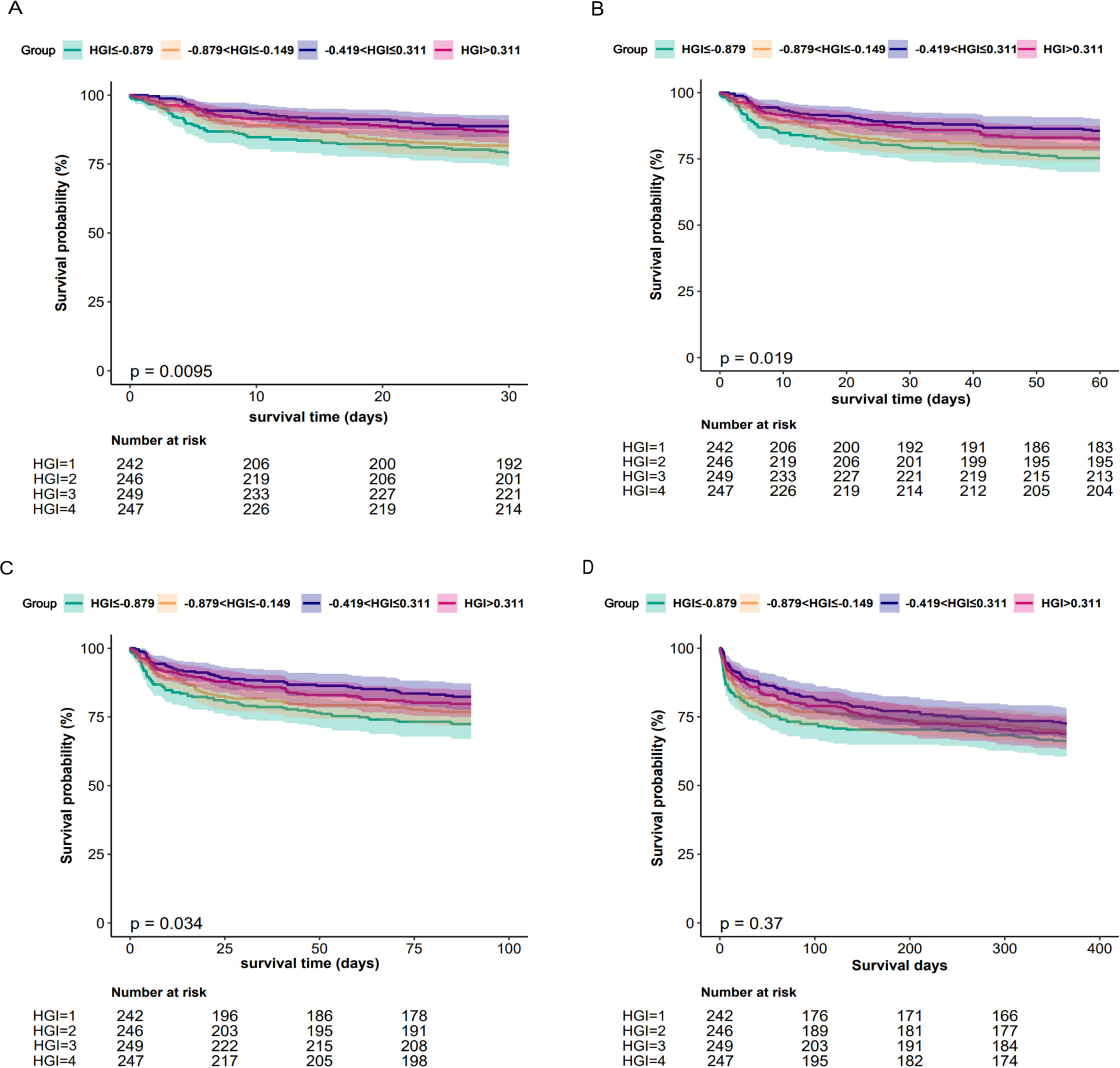
Kaplan-Meier all-cause mortality survival analysis curve **(A)** showing comparison of mortality within 30 days between groups, **(B)** showing comparison of mortality within 60 days between groups, **(C)** showing comparison of mortality within 90 days between groups, **(D)** showing comparison of mortality within 365 days between groups.

We compared the incidence of the primary outcome between groups using K-M survival analysis curves based on the HGI quartiles (as shown in **Figure 2**). The rate of mortality within 30 days or 60 days and 90 days was significantly higher in the Q1 group than Groups Q2 or Q3 and Q4 and the difference between the groups was significant (log-rank P<0.05) (**Figures 2A–C**), which indicated that low HGI were detrimental to the long-term survival of patients with CHF. The one-year mortality rate was also significantly higher in Groups Q1 than that in the other group, but no difference between the groups was significant (log-rank P>0.05) (**Figure 2D**).

### RCS regression analysis of HGI with one-year all-cause mortality

**Figure 3:**
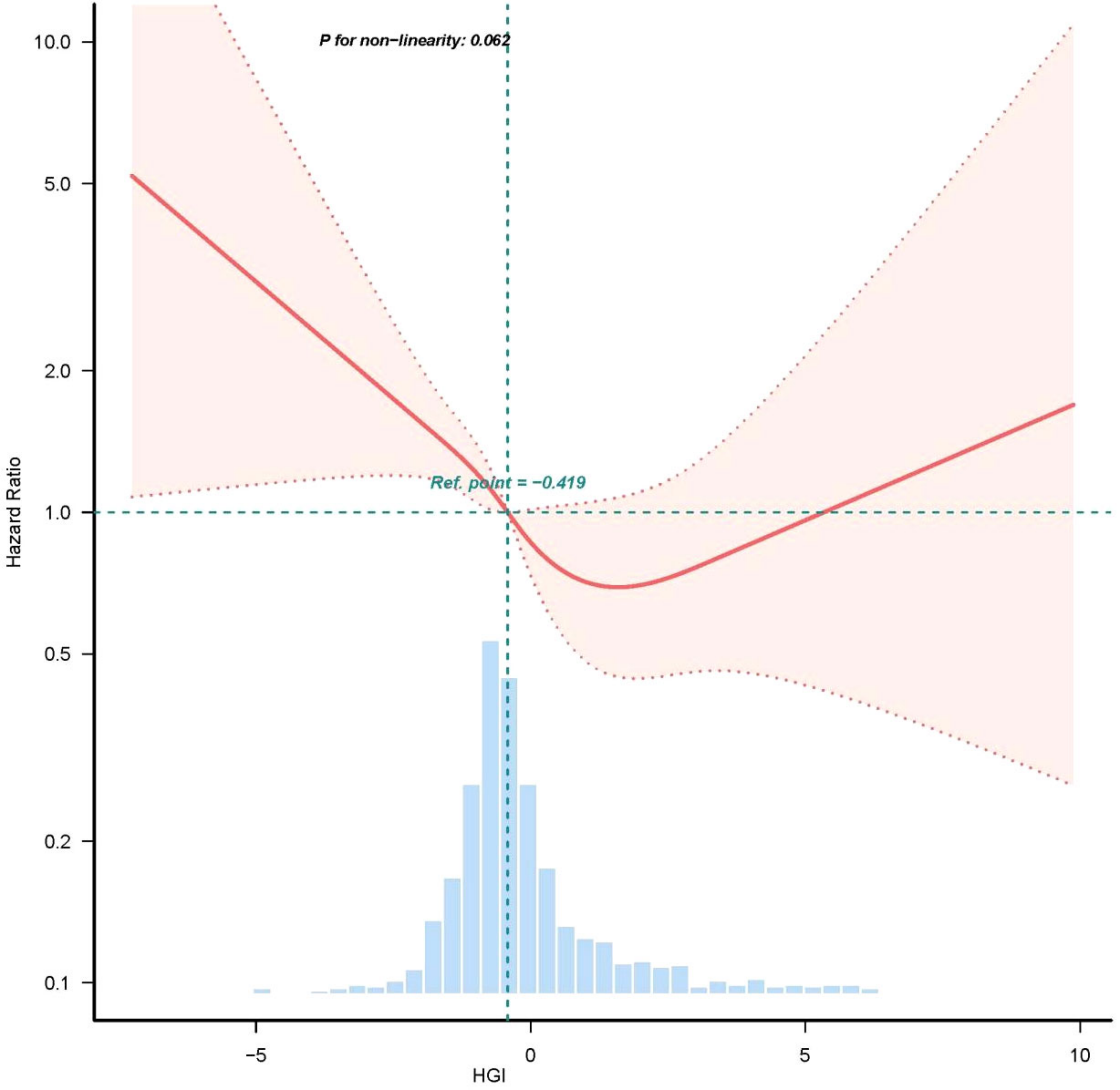
Restricted cubic spline curve for HGI hazard ratio. Vertical dashed lines indicate inflection points, dark gray lines indicate fully adjusted risk ratios, shaded areas indicate 95% confidence intervals, and horizontal dashed hazard ratio 1.

We employed RCS to further analyze the association between HGI and all-cause mortality in patients with CHF. The results indicated a “U-shaped” correlation between HGI and the risk of death in patients with severe CHF (**Figure 3**). In the threshold effect analysis, we identified the inflection points for HGI the primary outcomes to be -0.419.

### ROC curve analysis of HGI with CHF patient all-cause mortality

**Figure 4:**
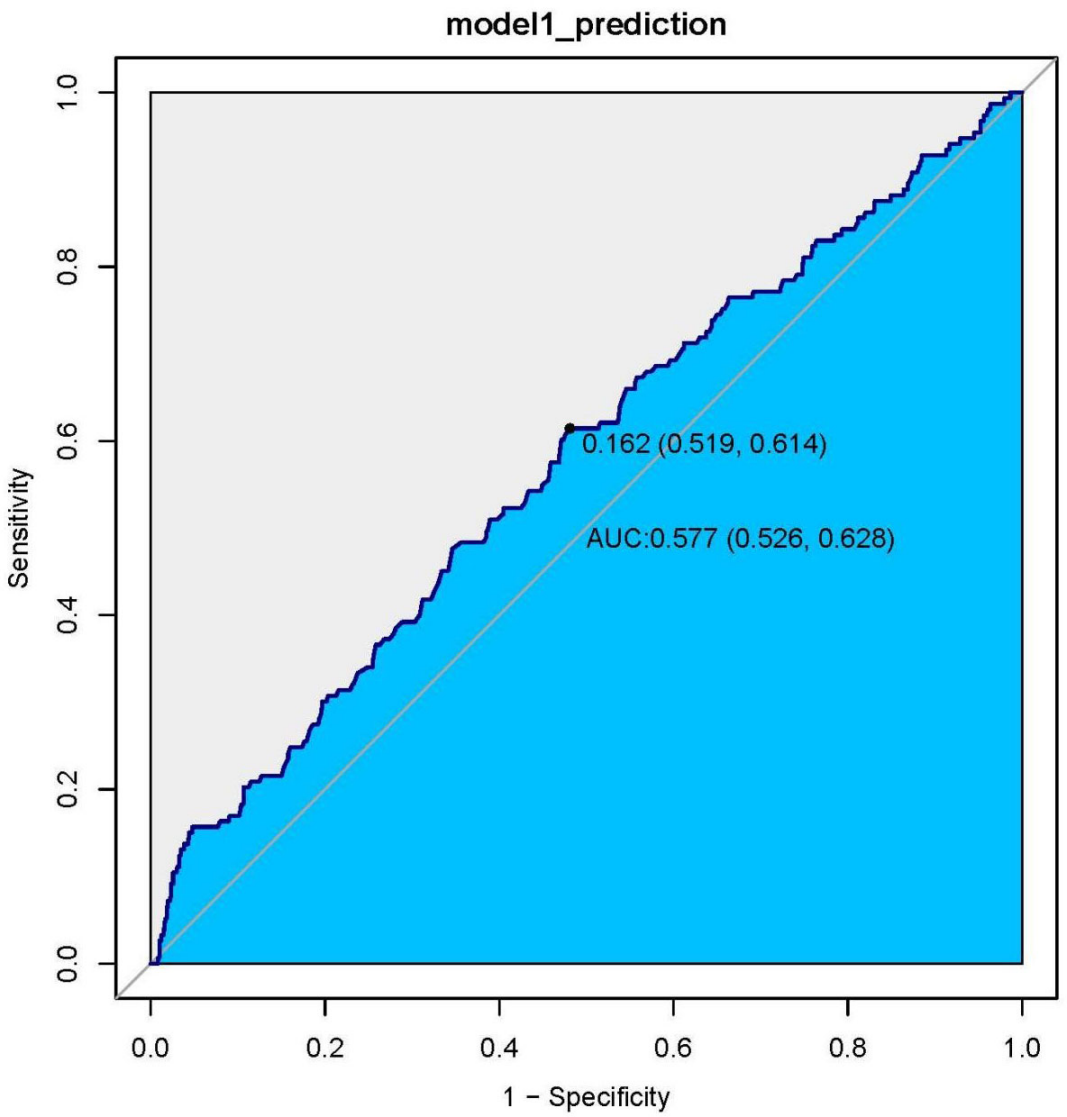
ROC curve analysis of HGI with CHF patient all-cause mortality.

We generated ROC curves and found the prognostic value of the HGI in all patients with CHF. The optimal cut-off value of the HGI in predicting death was 0.162, with an area under the curve of 0.577 (95% CI: 0.526–0.628), a sensitivity of 61.4% and a specificity of 51.9% (**Figure 4**).

### Subgroup analysis

We performed risk subgroup analyses of patient outcome events according to age, gender, diabetes and non-diabetes, cerebrovascular disease and non-cerebrovascular disease, use insulin and non-use insulin. Diabetes and non-diabetes. Use insulin and non-use insulin were significantly associated with with HGI in CHF. However, we observed that in a subgroup analysis with mortality as the outcome event, the four levels of HGI in the remaining subgroups were no statistically significant difference with primary 30 days, 90 days and 365 days mortality in patients with critical CHF. (**Supplementary Figure S1**).

## Discussion

A multitude of studies have demonstrated that the HGI is associated with a negative prognosis in cardiovascular diseases (CVDs), particularly in cases of coronary heart disease (CHD) and arrhythmia (19–21). While previous research has established a link between elevated HGI levels and an increased risk of CVD (22–24), evidence regarding the association between HGI and mortality in patients with congestive heart failure (CHF) remains inadequate. Therefore, this study developed a linear regression model utilizing MIMIC-IV data to evaluate the HGI in individuals with severe CHF and to investigate the relationship between HGI and adverse outcomes in these patients.

The data we gathered indicated that the mortality rate among patients was 15.9% within the initial 30 days of hospital admission, rising to 19.3% by 60 days, 22.1% at 90 days, and escalating to 30.7% by 365 days post-admission. In the fully adjusted Cox proportional hazards model, HGI demonstrated a significant correlation with mortality rates at both the 30-day and 60-day intervals for patients suffering from severe CHF. An elevated HGI functions as a protective factor, enhancing the survival outlook for individuals with CHF. These findings could be instrumental in developing management strategies for CHF patients.

In our evaluation rooted in the RCS curve analysis, a ‘U’-shaped connection has been identified between the HGI and the risk ratio for outcome events, with inflection points nearing zero, aligning with earlier research (13, 25). Within a specific cohort, HbA1c levels exceeding 10% demonstrate a progressively non-linear association with HF development, while levels falling below 6% have been linked to a heightened risk of HF (1). Currie et al. previously undertook a retrospective analysis of T2DM patients via the UK General Practice Research Database (GPRD) (26, 27) revealing a U-shaped correlation between average HbA1c levels and overall mortality as well as cardiac incidents. A recent investigation has indicated that patients exhibiting low HbA1c alongside high glycemic variability show poorer left ventricular diastolic function than those with elevated HbA1c but low glycemic variability (28). Inadequate or subpar blood glucose management can lead to microvascular changes and cardiac fibrosis, increasing the HF risk (29). These observations imply that both elevated and diminished HbA1c values can forecast HF progression. Consequently, it is essential in clinical practice to develop a linear regression model to compute the HGI for a substantial patient population.

Previous studies have identified several risk factors for the development of HF in T2DM, including increasing age, body mass index (BMI), SBP, baseline creatinine levels, and a history of coronary artery disease (CAD) (30, 31). Our univariate analysis indicates that age, race, MI, cerebrovascular disease, DM, paraplegia, renal disease, CCI, SBP, respiratory rate, WBC, RBC, and creatinine are risk factors for heart rate (HR). Even after multivariate adjusting the above risk factors, Medium to high level HGI was still independently related to mortality in CHF. Additionally, our cox regression analysis suggest that SBP is one of the risk factors for HF patients. Specifically, maintaining lower SBP levels is significantly linked to a reduced risk of acute MI and stroke in this population (32). Moreover, SBP has been correlated with a reduced risk of cardiovascular events and mortality (33). The Systolic Blood Pressure Intervention Trial (SPRINT) provided further evidence, illustrating that targeting SBP levels below the standard guidelines for non-diabetic individuals can lead to a decreased risk of cardiovascular outcomes and death (34). Lebeche et al. indicates that elevated HGI levels correspond with a greater prevalence of HF in hypertensive patients (35). Consequently, HGI may represent an independent risk factor for individuals experiencing severe CHF.

Our analysis of subgroups indicates that patients with HF who also have diabetes may experience a more pronounced protective effect from higher levels of the HGI compared to HF patients without diabetes. In individuals with HF, the presence of DM has been shown to independently correlate with increased overall mortality, cardiovascular deaths, and a higher frequency of hospital readmissions (36, 37). Most evidence suggests that hyperglycemia, regardless of the treatment administered, can alter the quantitative relationship between HbA1c and blood glucose levels, resulting in a significant increase in HGI levels (38–40). Consequently, diabetic patients are more likely to exhibit elevated HGI levels and may benefit from improved glycemic control interventions. This observation suggests that the HGI could hold greater prognostic significance for patients suffering from advanced heart failure who do not have diabetes.

The study presents several limitations that warrant acknowledgment. Firstly, BMI and other factors have not been measured or may have been inadequately assessed, which could still affect the associations we observed. Secondly, we did not implement a time-dependent model to analyze the use of antihyperglycemic medications. As a result, we are unable to ascertain whether the relationship between these medications and the progression of heart failure (HF) is influenced by factors. Lastly, while the study included data on all-cause mortality, we did not collect information specifically regarding cardiovascular mortality or hospitalizations due to heart failure. This gap highlights the need for further investigation to uncover more nuanced insights into the causes of mortality within this patient demographic.

In conclusion, by utilizing patient data retrieved from the MIMIC-IV database, the authors were able to demonstrate a nonlinear relationship HGI and all-cause mortality in patients with CHF. A higher HGI has a more protective effect than a low HGI for patients with CHF and is closely related to both short-term mortality rates. We believe that HGI is a good prognostic indicator for poor outcomes in CHF patients and could serve as a potential marker for stratifying the risk of short-term and long-term mortality in this patient population. These findings may aid in the management of CHF patients and their blood sugar levels and directing limited healthcare resources towards those who require more intensive management.

## Data Availability

The datasets presented in this study can be found in online repositories. The names of the repository/repositories and accession number(s) can be found below: No applicable.
