# Supplementary material for: Association between Hemoglobin Glycation Index and In-Hospital all-cause mortality of patients with Congestive Heart Failure: a retrospective study utilizing the MIMIC-IV database
Source: Front Endocrinol (Lausanne). 2025 Mar 19;16:1475063. doi: 10.3389/fendo.2025.1475063 (PMC11986639; doi:10.3389/fendo.2025.1475063)
Supplement: Supplementary file 1 [file Image1.pdf]

Subgroup	Variable	Total	Event (%)	HR (95%CI)	P for interaction
Age≥45	HGI	76	6 (7.9)	0.72 (0.33~1.57)	0.86
Age<45	HGI	909	151 (16.6)	0.87 (0.77~0.99)	
Gender=male	HGI	579	80 (13.8)	0.84 (0.71~0.99)	0.958
Gender=female	HGI	406	77 (19)	0.97 (0.81~1.16)	
Race=1	HGI	609	89 (14.6)	0.92 (0.77~1.09)	0.716
Race=2	HGI	97	9 (9.3)	0.94 (0.58~1.52)	
Race=3	HGI	52	6 (11.5)	0 (0~Inf)	
Race=4	HGI	227	53 (23.3)	0.89 (0.72~1.1)	
Diabetes=no	HGI	542	89 (16.4)	0.54 (0.4~0.75)	<0.001
Diabetes=yes	HGI	443	68 (15.3)	0.98 (0.85~1.12)	
cerebrovascular disease=no	HGI	665	76 (11.4)	0.95 (0.82~1.1)	0.409
cerebrovascular disease=yes	HGI	320	81 (25.3)	0.79 (0.62~1)	
use insulin=no	HGI	368	45 (12.2)	0.56 (0.33~0.95)	0.019
use insulin=yes	HGI	617	112 (18.2)	0.91 (0.8~1.03)	

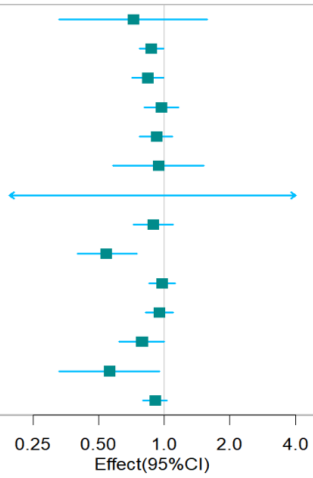

Supplementary Fig.1a Forest plot of subgroup analysis 30 days mortality analysis with HGI classification in CHF patients. HGI: haemoglobin glycation index; CHF: congestive heart failure CI: confidence interval; HR: hazard ratio.

Subgroup	Variable	Total	Event (%)	HR (95%CI)	P for interaction
Age≥45	HGI	76	9 (11.8)	0.75 (0.34~1.64)	0.829
Age<45	HGI	909	209 (23)	0.91 (0.82~1)	
Gender=male	HGI	579	107 (18.5)	0.85 (0.74~0.98)	0.633
Gender=female	HGI	406	111 (27.3)	1 (0.86~1.16)	
Race=1	HGI	609	121 (19.9)	0.97 (0.83~1.12)	0.306
Race=2	HGI	97	20 (20.6)	0.88 (0.62~1.27)	
Race=3	HGI	52	8 (15.4)	0.21 (0.04~0.98)	
Race=4	HGI	227	69 (30.4)	0.92 (0.76~1.1)	
Diabetes=no	HGI	542	125 (23.1)	0.66 (0.5~0.88)	0.008
Diabetes=yes	HGI	443	93 (21)	0.97 (0.87~1.09)	
cerebrovascular disease=no	HGI	665	105 (15.8)	0.95 (0.83~1.08)	0.609
cerebrovascular disease=yes	HGI	320	113 (35.3)	0.83 (0.68~1.01)	
use insulin=no	HGI	368	64 (17.4)	0.74 (0.47~1.14)	0.131
use insulin=yes	HGI	617	154 (25)	0.92 (0.83~1.03)	

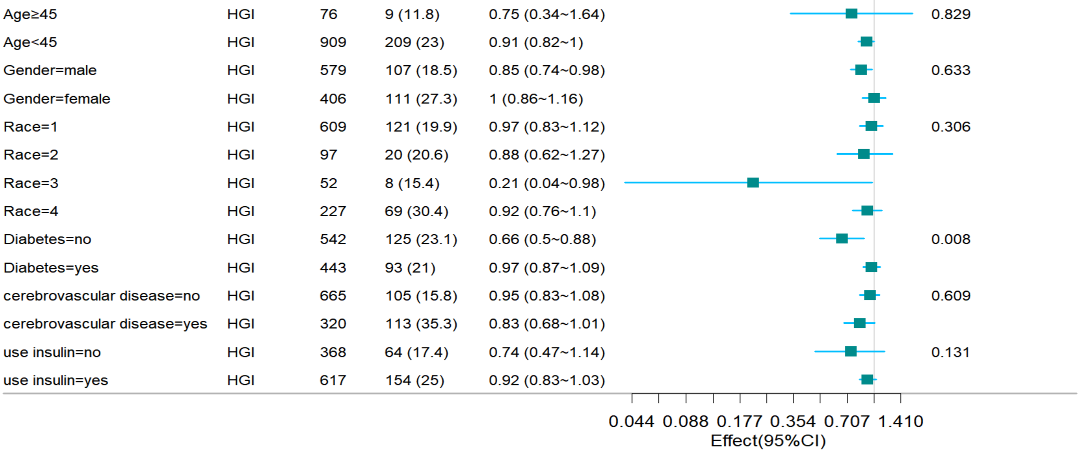

Supplementary Fig.1b Forest plot of subgroup analysis 90 days mortality analysis with HGI classification in CHF patients. HGI: haemoglobin glycation index; CHF: congestive heart failure CI: confidence interval; HR: hazard ratio.

Subgroup	Variable	Total	Event (%)	HR (95%CI)	P for interaction
Age≥45	HGI	76	11 (14.5)	1.02 (0.72~1.45)	0.728
Age<45	HGI	909	291 (32)	0.97 (0.89~1.06)	
Gender=male	HGI	579	157 (27.1)	0.97 (0.87~1.08)	0.604
Gender=female	HGI	406	145 (35.7)	1.01 (0.89~1.15)	
Race=1	HGI	609	183 (30)	1.02 (0.91~1.14)	0.19
Race=2	HGI	97	28 (28.9)	1.04 (0.83~1.3)	
Race=3	HGI	52	9 (17.3)	0.49 (0.18~1.35)	
Race=4	HGI	227	82 (36.1)	0.95 (0.81~1.12)	
Diabetes=no	HGI	542	166 (30.6)	0.88 (0.69~1.13)	0.273
Diabetes=yes	HGI	443	136 (30.7)	1.02 (0.93~1.11)	
cerebrovascular disease=no	HGI	665	160 (24.1)	1.01 (0.92~1.12)	0.383
cerebrovascular disease=yes	HGI	320	142 (44.4)	0.87 (0.73~1.04)	
use insulin=no	HGI	368	95 (25.8)	1.19 (0.83~1.7)	0.497
use insulin=yes	HGI	617	207 (33.5)	0.98 (0.9~1.07)	

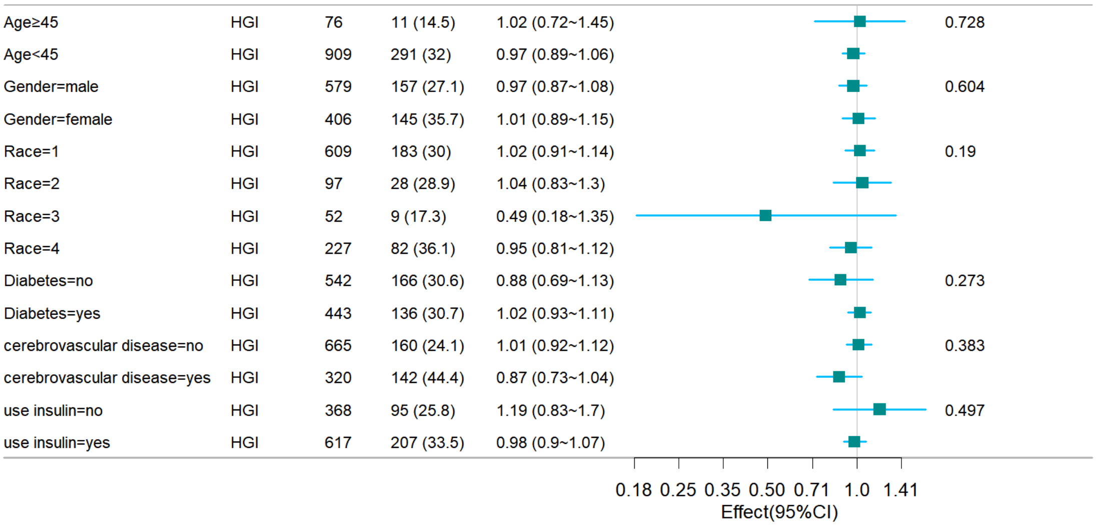

Supplementary Fig.1c Forest plot of subgroup analysis 365 days mortality analysis with HGI classification in CHF patients. HGI: haemoglobin glycation index; CHF: congestive heart failure CI: confidence interval; HR: hazard ratio.

Figure Legends

Supplementary Figure.1 a Forest plot of subgroup analysis 30 days mortality analysis with HGI classification in CHF patients. b Forest plot of subgroup analysis 90 days mortality analysis with HGI classification in CHF patients. c. Forest plot of subgroup analysis 365 days mortality analysis with HGI classification in CHF patients.
